# Chitosan Oligosaccharides Attenuate Ocular Inflammation in Rats with Experimental Autoimmune Anterior Uveitis

**DOI:** 10.1155/2014/827847

**Published:** 2014-07-24

**Authors:** I-Mo Fang, Chang-Hao Yang, Chung-May Yang

**Affiliations:** ^1^Department of Ophthalmology, Taipei City Hospital, Zhongxiao Branch, Taipei, Taiwan; ^2^Department of Ophthalmology, National Taiwan University Hospital, No. 7, Chung-Shan S. Road, Taipei 10041, Taiwan

## Abstract

We investigated the protective effects and mechanisms of chitosan oligosaccharides (COS) on experimental autoimmune anterior uveitis (EAAU) in rats. EAAU was induced in Lewis rats by footpad and intraperitoneal injections of melanin-associated antigen. The rats received intraperitoneal injections of low-dose (5 mg/kg) or high-dose (10 mg/kg) COS or PBS daily after the immunization. The effects of COS were evaluated by determining the clinical scores and the morphology of the iris/ciliary body (ICB). The expression of inflammatory mediators was evaluated using western blot, immunofluorescence, and ELISA. Treatment with COS significantly attenuated the clinical scores and the leukocyte infiltration in the ICB in a dose-dependent manner. COS effectively reduced the expression of inflammatory mediators (TNF-*α*, iNOS, MCP-1, RANTES, fractalkine, and ICAM-1). Moreover, COS decreased the I*κ*B degradation and p65 presence in the ICB, which resulted in the inhibition of NF-*κ*B/DNA binding activity. In an in vitro study, sensitized spleen-derived lymphocytes of the COS-treated group showed less chemotaxis toward their aqueous humor and decreased secretion of the above inflammatory mediators in the culture media. COS treated EAAU by inhibiting the activation of NF-*κ*B and reducing the expression of inflammatory mediators. COS might be a potential treatment for acute anterior uveitis.

## 1. Introduction

Acute anterior uveitis (AAU) is the most common uveitis in humans. AAU can cause significant visual problems because of its recurrent nature and might result in secondary complications, such as cataract formation, cystoid macular edema, and glaucoma [1]. The exact mechanism of AAU remains unknown. Topical corticosteroids are generally the mainstay in the treatment of AAU; however, periocular injections and systemic steroids are necessary in recalcitrant cases. The long-term use of corticosteroids might produce a wide range of systemic and ocular side effects [2]. Therefore, there is increasing interest in therapies with new molecules that eliminate the side effects of corticosteroids but are as efficient in reducing ocular inflammation and preventing tissue destruction.

Experimental autoimmune anterior uveitis (EAAU), an animal model of human acute anterior uveitis, can be induced in Lewis rats by immunization with bovine melanin-associated antigen (MAA) [3–5]. The clinical course and pathology observed in EAAU are strikingly similar to the related processes in human AAU. The inflammation, which is confined exclusively to the anterior segment without retinal and choroid involvement, differs greatly from other uveitis models [6–8]. This characteristic makes EAAU the best model of human acute anterior uveitis.

Chitosan oligosaccharides (COS), the hydrolyzed product of chitosan, are a mixture of oligomers of *β*-1,4-linked D-glucosamine residues and are abundant in the exoskeleton of crustaceans and in the cell walls of fungi and insects [9]. COS are known to have various biological effects, including antitumor, antibacterial, anti-inflammation, antioxidative, and antiapoptotic activities [10–13]. COS are nontoxic and biodegradable and have been used as a bioactive material. In addition, COS have good solubility in water and are easily absorbed in the intestine, making them an attractive ingredient in many healthy foods or dietary supplements. We previously showed that COS exerted antioxidative effects by inhibiting NF-*κ*B activation and attenuating oxidative-stress related retinal degeneration in rats [14]. However, the effects of COS for the anti-inflammatory activity in a rat model of EAAU remain unknown.

In this study, we investigated the therapeutic effects and possible mechanisms of COS in EAAU rats. Furthermore, an in vitro study using sensitized spleen-derived lymphocytes was performed to verify the possible mechanisms of COS action.

## 2. Materials and Methods

### 2.1. Reagents

Chitosan oligosaccharides and paraquat were purchased from Sigma-Aldrich (St. Louis, MO, USA). A green fluorescent protein (GFP) antibody was purchased from BioVision (Mountain View, CA, USA). Mounting medium with 4′,6-diamidino-2-phenylindole (DAPI) was obtained from Vector Laboratories (Burlingame, CA, USA). The anti-rat TNF-*α*, MCP-1, and RANTES antibodies were purchased form Peprotech (Rocky Hill, NJ, USA), the anti-rat fractalkine antibody was from eBioscience (San Diego, CA, USA), and the anti-p65 antibody was from Rockland (Gilbertsville, PA, USA). The MCP-1 and RANTES ELISA kits were obtained from Peprotech. The TNF-*α* ELISA kit was from BioLegend (San Diego, CA, USA) and the fractalkine ELISA kit was from R&D Systems (Minneapolis, MN, USA). The nitric oxide (NO) ELISA kit was purchased from Cayman (Ann Arbor, MI, USA).

### 2.2. Antigen Preparation and Induction of EAAU

Melanin-associated antigen (MAA) was prepared as described by Broekhuyse et al. with a modification [4]. The iris and ciliary body were carefully obtained from freshly pigmented bovine eyes. The tissue was homogenized gently and filtered through a wire mesh to remove the cellular debris and connective tissue. The homogenate was centrifuged at 1.2 × 10^5^ g at 4°C for 15 min and washed once with PBS (pH 7.4). The resulting pellet was resuspended in 2% SDS and incubated at 70°C for 10 min. After centrifugation, the pellet was washed three times in water. The insoluble antigen was dried and stored at −20°C.

Lewis rats, 6–8-week old and weighing 125–160 g, were used for the experiment. All animals were treated in accordance with the ARVO statement for the Use of Animals in Ophthalmic and Vision Research. To induce EAAU, the rats were given two separate injections simultaneously. The first injection was 0.05 mL MAA, suspended in PBS, emulsified (1 : 1) in complete Freund's adjuvant (Sigma-Aldrich), and injected into the left hind footpad and the second injection was 0.05 mL MAA, emulsified with 1 *μ*g purified Bordetella pertussis toxin (List, Campbell, CA, USA) and injected intraperitoneally.

### 2.3. Animal Grouping and Treatment

The experimental rats were randomly divided into four groups.

 Group 1: rats received footpad and intraperitoneal injections of PBS as the normal control (normal).
 Group 2: rats received daily intraperitoneal injections of PBS, beginning immediately after footpad and intraperitoneal injections of MAA to induce EAAU (PBS-treated group).
 Group 3: rats received daily intraperitoneal injections of 5 mg/kg COS, beginning immediately after footpad and intraperitoneal injections of MAA to induce EAAU (low-dose COS group).
 Group 4: rats received daily intraperitoneal injections of 10 mg/kg COS, beginning immediately after footpad and intraperitoneal injections of MAA to induce EAAU (high-dose COS group).

In the preliminary study, we have tried 4 doses of COS (5, 10, 20, or 50 mg/kg) for intraperitoneal injections daily into the rats after immunization. However, the rats receiving 20 or 50 mg/kg COS injections became of poor appetite, decreased activity, and increased abdominal circumference after daily injections for 10 days. These rats became emaciated with distended abdomens and some rats even died after continued injections of 20 or 50 mg/kg COS. These findings indicated that intraperitoneal injections of COS at the concentrations of 20 or 50 mg/kg would cause severe toxic effects. Therefore, we chose 5 or 10 mg/kg COS to treat rats with EAAU in our study.

Total numbers of animals used at definite time points in each group and the days to perform the experiments were summarized in Table 1.

### 2.4. Clinical Examination

The rats were clinically observed each day using slit lamp biomicroscopy for clinical signs of ocular inflammation. The disease severity was clinically assessed with a scale ranging from 0 to 4: 0 = normal; 1 = slight iris-vessel dilatation and some anterior chamber cells; 2 = iris hyperemia, with some limitation in pupil dilation, anterior chamber cells, and a slight flare; 3 = miotic, irregular, hyperemic, and slightly damaged iris, with a considerable flare and cells (especially with accumulation near the iris); 4 = a seriously damaged and hyperemic iris, a miotic pupil often filled with protein, and cloudy gel-like aqueous humor (AqH).

### 2.5. Histological Evaluation of the Anterior Segment and Quantification of Leukocytes in the AqH

In a separate experiment, three rats in each group were randomly selected and sacrificed at days 10, 14, 17, and 20, respectively. Aqueous humor (2 *μ*L) was obtained from each rat using anterior chamber paracentesis with a 30-gauge needle. The sample was stained with 0.4% trypan blue and then observed under phase contrast microscopy to calculate the number of leukocytes.

The eyeballs were enucleated and immersed in 4% paraformaldehyde in 0.2 M phosphate buffer for 24 h. After fixation, the eyes were dehydrated with alcohol and embedded in paraffin. The specimens were cut into 5 *μ*m sagittal sections near the optic nerve head and were stained with hematoxylin and eosin (H&E) to evaluate the cellular infiltration in the iris and ciliary body (ICB).

### 2.6. Western Blot Analysis

The rats of each group were sacrificed at day 14. The total protein was extracted from the ICB by lysing the sample in radioimmunoprecipitation assay (RIPA) buffer (0.5 M Tris-HCl (pH 7.4), 1.5 M NaCl, 2.5% deoxycholic acid, 10% NP-40, 10 mM EDTA, and protease inhibitors). The extract and Laemmli buffer were mixed at a 1 : 1 ratio, and the mixture was boiled for 5 min. The samples (100 *μ*g) were separated on 10% SDS-polyacrylamide gels and transferred to polyvinylidene difluoride membranes (Millipore, Billerica, MA, USA). The membranes were incubated with anti-TNF-*α*, anti-iNOS, anti-MCP-1, anti-RANTES, anti-fractalkine, anti-ICAM-1, anti-I*κ*B, anti-NF-*κ*B p65, and anti-*β*-actin antibodies. The membranes were then incubated with a horseradish peroxidase-conjugated secondary antibody and visualized using chemiluminescence (GE Healthcare, Buckinghamshire, UK). The density of the blots was quantified using image-analysis software (Photoshop, version 7.0; Adobe Systems, San Jose, CA, USA). The optical densities of each band were calculated and standardized based on the density of the *β*-actin band.

### 2.7. Immunohistochemical (IHC) Studies

Three rats in each group were used for immunohistochemical studies. Immunohistochemistry was carried out by simultaneously blocking and permeabilizing sections with 0.2% Triton in PBS containing 5% goat serum for 1 hr at room temperature, incubating with TNF-*α*, iNOS, MCP-1, RANTES, fractalkine, ICAM-1, and NF-*κ*B p65 primary antibodies diluted in blocking solution overnight at 4°C, and incubating with the appropriate fluorescent secondary antibodies (all diluted 1 : 1000) in blocking solution for 3 hrs at room temperature. The nuclei were counterstained with DAPI.

The following formula was used for the densitometric quantitation of TNF-*α*, iNOS, MCP-1, RANTES, fractalkine, ICAM-1, and NF-*κ*B p65, as previously described [15] with modification:

(1) Immunostaining  index =∑[(X−threshold)×area(pixels)]total  cell  number,

where *X* is the staining density indicated by a number between 0 and 256 in grayscale, and *X* is more than the threshold. Briefly, digitized color images were obtained as PICT files. PICT files were opened in grayscale mode using NIH image, version 1.61. Cell numbers were determined using the Analyze Particle command after setting a proper threshold.

The relative density of immunostaining was defined as immunostaining index of PBS-treated, low-dose, or high-dose COS groups divided by immunostaining index of normal group.

### 2.8. Quantification of the Levels of TNF-*α*, Nitric Oxide (NO), MCP-1, RANTES, and Fractalkine in the AqH

Five rats in each group were sacrificed at days 10, 14, 17, and 20, respectively. The concentrations of TNF-*α*, NO, MCP-1, RANTES, and fractalkine were determined using commercial ELISA kits according to the manufacturers' instructions. The AqH (5 *μ*L) were diluted to 50 *μ*L for testing, and the optical density was determined at A_450_ (absorbance at 450 nm) using a microplate reader (Bio-Rad).

### 2.9. Nuclear Protein Extract and Electrophoretic Mobility Shift Assay of NF-*κ*B (EMSA)

Five rats in each group were sacrificed at day 14. The ICB was minced in 0.5 mL of ice-cold buffer A containing 10 mM HEPES (pH 7.9), 1.5 mM KCl, 10 mM MgCl_2_, 1.0 mM dithiothreitol (DTT), and 1.0 mM phenylmethylsulfonyl fluoride (PMSF). The tissue was homogenized, followed by centrifugation at 5000 g at 4°C for 10 min. The sediment was suspended in 200 *μ*L of buffer B. The suspension was incubated on ice for 30 min. The sample was centrifuged at 12,000 g at 4°C for 30 min. The supernatant containing the nuclear proteins was collected. The EMSA was performed using an NF-*κ*B DNA-binding protein-detection system (Pierce Biotechnology, Rockford, IL, USA). A 10 *μ*g nuclear protein aliquot was incubated in binding buffer with a biotin-labeled NF-*κ*B consensus oligonucleotide probe (5′-AGTTGAGGGGACTTTCCCAG-GC-3′) for 30 min and resolved in 6% nondenaturing polyacrylamide gel. The specificity of the DNA/protein binding was determined by adding a 100-fold molar excess of unlabeled NF-*κ*B oligonucleotide for competitive binding 10 min before adding the biotin-labeled probe. The protein-DNA-biotin complexes were blotted onto a nitrocellulose transfer membrane followed by UV cross-linking. The complexes were revealed with streptavidin-horseradish peroxidase conjugate and SuperSignal chemiluminescent substrate and then exposed to X-ray film. The density of the blots was quantified using image-analysis software (Photoshop). “Fold change” was defined as optic density of the PBS-treated, low-dose, or high-dose COS group divided by optic density of the normal group.

### 2.10. Cell Preparation of Spleen-Derived Lymphocytes

Single cell suspensions from the spleens of the four groups at day 14 were prepared by mashing the tissues with frosted slides (Fisher Scientific), followed byfiltration through a cell strainer (BD Biosciences, SanJose, CA, USA). The red blood cells were removed by treating the cells with red blood cell lysis buffer (2.07 g NH_4_Cl, 0.25 g NaHCO_3_, and 9.3 mg EDTA in 100 mL H_2_O). The total lymphocytes werepurified by passing the cells through a Histopaque-1077 gradient (Sigma-Aldrich) according to the manufacturer's protocol. The cells were suspended in complete RPMI 1640 culture medium with L-glutamine containing 1% (v/v) minimum essential medium (Life-Gibco, Rockville, MD, USA), NEAA (BioWhittaker, Allendale, NJ, USA), a mixture of antibiotics (100 U/mL penicillin, 100 U/mL streptomycin, and 0.25 *μ*g/mL Amphotericin B), and 10% (v/v) fetal bovine serum (Life-Gibco). The cells of each group were exposed to 10 *μ*g/mL MAA for 3 days.

### 2.11. Lymphocyte Chemotaxis Assay

Lymphocyte chemotaxis was measured using QCM chemotaxis 96-well plates fitted with 3 mm membrane inserts (Millipore). The lymphocytes of the four groups were placed in the upper chamber of a QCM apparatus, and the aqueous humor samples of the same groups were placed in the lower chamber of the QCM apparatus. After 24 hrs of incubation, the cells that migrated toward the chemoattractant were recovered from the lower chamber, and the unmigrated cells were recovered from the inserts. The migrated cells were stained with a green fluorescent dye (CyQuant GR dye, Millipore) and transferred to a 96-well flat-bottomed ELISA microplate; the fluorescence was measured at 485/535 nm using a plate reader (Perkin Elmer, Waltham, MA, USA). The data are reported in fluorescent units representing cells that migrated into the lower chamber toward the chemoattractant.

### 2.12. Evaluation of the Levels of TNF-*α*, MCP-1, RANTES, and Fractalkine in Culture Media of Spleen-Derived Lymphocytes

The culture media of spleen-derived lymphocytes of each group were obtained. The levels of TNF-*α*, MCP-1, RANTES, and fractalkine in the culture media were measured using ELISA kits as described previously.

### 2.13. Statistical Analysis

The results are expressed as the mean ± SD. To compare the numerical data among four groups, Kurskal-Wallis *H* test followed by post hoc Dunn test was used. A *P* value of 0.05 or less was considered significant. All of the data were analyzed using SPSS 10.0.

## 3. Results

### 3.1. Effects of COS on Clinical Activity Scores

**Figure 1:**
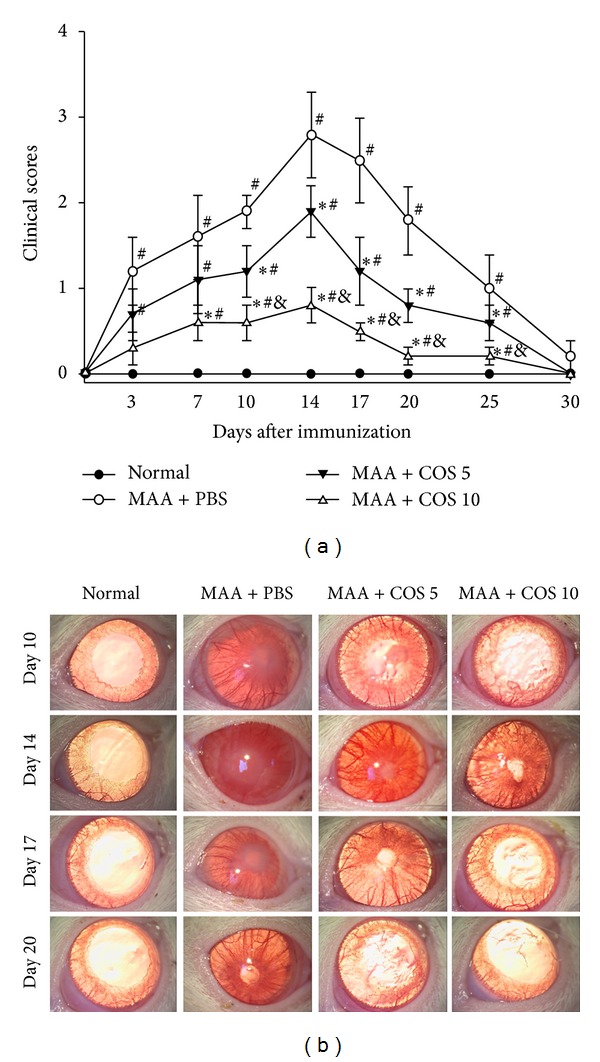
(a) Effects of COS on ocular inflammation demonstrated by clinical scores. The rats in each group were clinically observed on a daily basis using slit lamp biomicroscopy for clinical signs of ocular inflammation. Disease severity was clinically assessed with a scale ranging from 0 to 4. The data are expressed as the mean ± SD (^∗^ *P* < 0.05 compared with the PBS-treated group; ^#^ *P* < 0.05 compared with the normal group; ^&^ *P* < 0.05 compared with the low-dose group by Kruskal-Wallis *H* test with post hoc Dunn test; *n* = 10 for each group). (b) Representative clinical photographs of the four groups at days 10, 14, 17, and 20 are shown.

The rats induced with MAA began to develop signs of EAAU on day 3 after immunization. The clinical signs reached a peak at day 14 and were entirely relieved at day 30. Treatment with low-dose COS caused a significant reduction in the clinical activity scores at days 10, 14, 17, and 20 (*P* < 0.05 in all paired comparisons, *n* = 10). Moreover, the high-dose COS group demonstrated significantly decreases in the clinical activity scores throughout the clinical course, at days 7, 10, 14, 17, 20, and 25, compared with the PBS-treated group (*P* < 0.05 in all paired comparisons, *n* = 10). The clinical activity scores were significantly lower in the rats treated with high-dose COS than in the rats in the low-dose COS group at days 10, 14, 17, 20, and 25 (*P* < 0.05 in all paired comparisons, *n* = 10) (Figures 1(a) and 1(b)).

### 3.2. Effects of COS on Histological Changes in ICB and Leukocytes Infiltration in AqH

**Figure 2:**
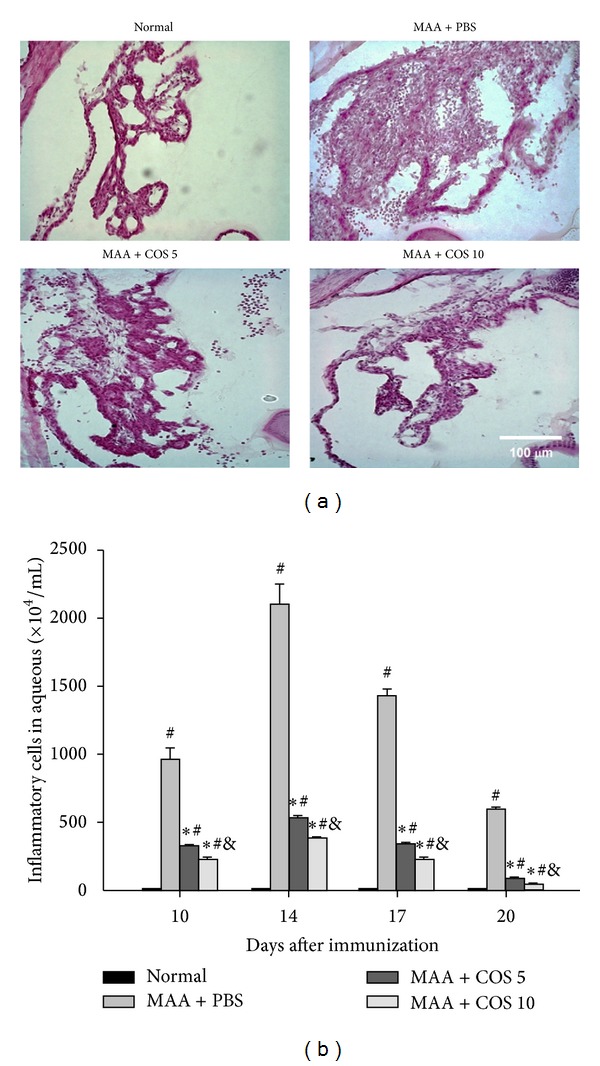
Effects of COS on histological changes in the iris/ciliary bodies (ICB) and leukocyte infiltration in the aqueous humor (AqH). (a) A representative H&E-stained ICB from normal or PBS-treated or high- or low-dose COS groups at day 14. The EAAU rats showed increased leukocyte infiltration in the ICB and the AqH, and the inflammation was attenuated by treatment with low-dose or high-dose COS. Original magnification 100x. (b) The quantification of leukocytes in the AqH at days 10, 14, 17, and 20. The EAAU rats treated with low-dose or high-dose COS showed significantly reduced leukocyte numbers in the AqH. The data are expressed as the mean ± SD (^∗^ *P* < 0.05 compared with the PBS-treated group; ^#^ *P* < 0.05 compared with the normal group; ^&^ *P* < 0.05 compared with the low-dose group by Kruskal-Wallis *H* test with post hoc Dunn test; *n* = 3 for each group).

The histological examination revealed that the PBS-treated group had prominently increased leukocyte infiltration and tissue swelling in the ICB at day 14. Treatment with low-dose or high-dose COS resulted in a markedly decreased infiltration of leukocytes in the ICB. The effects of attenuated leukocyte infiltration and tissue swelling in the ICB were more noticeable in the high-dose COS group than in the low-dose COS group (Figure 2(a)).

In the PBS-treated group, the number of leukocytes in the AqH significantly increased at days 10, 14, 17, and 20 compared with the normal group (*P* < 0.05 in all paired comparisons, *n* = 3). The number of leukocytes was significantly lower in the rats treated with low-dose or high-dose COS compared with the rats treated with PBS at days 10, 14, 17, and 20 (*P* < 0.05 in all paired comparisons, *n* = 3). In addition, the number of leukocytes was significantly reduced in the high-dose COS group compared with the low-dose COS treatment group at days 10, 14, 17, and 20 (*P* < 0.05 in all paired comparisons, *n* = 3) (Figure 2(b)).

### 3.3. Western Blot for the Effects of COS on the Expression of TNF-*α*, iNOS, MCP-1, RANTES, Fractalkine, and ICAM-1 in the ICB

**Figure 3:**
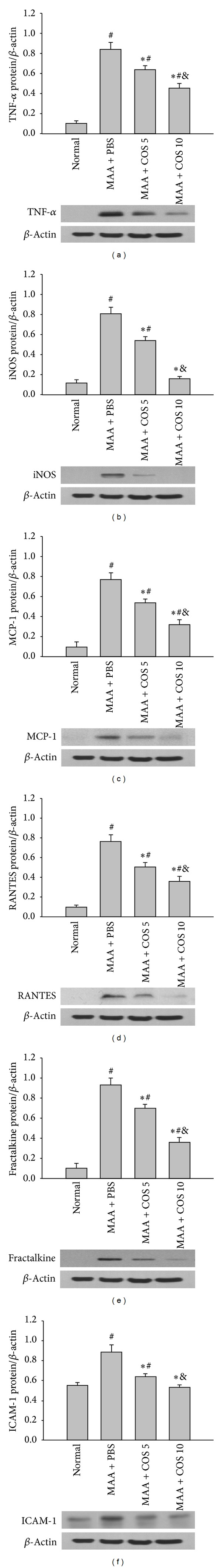
Evaluation of the protein expression levels of inflammatory mediators at day 14 by western blot analysis. The protein levels of (a) TNF-*α*, (b) iNOS, (c) MCP-1, (d) RANTES, (e) fractalkine, and (f) ICAM-1 were significantly higher in the PBS-treated group compared with the normal rats. In the COS-treated groups, especially in the high-dose group, the levels of inflammatory mediators were significantly lower than in the PBS-treated group. The data are expressed as the mean ± SD (^∗^ *P* < 0.05 compared with the PBS-treated group; ^#^ *P* < 0.05 compared with the normal group; ^&^ *P* < 0.05 compared with the low-dose group by Kruskal-Wallis *H* test with post hoc Dunn test; *n* = 8 for each group).

The expression levels of the TNF-*α*, iNOS, MCP-1, RANTES, fractalkine, and ICAM-1 proteins in the ICB were significantly higher in the PBS-treated group compared with the normal group at day 14 (*P* < 0.05 in all paired comparisons; *n* = 8). Treatment with low- or high-dose COS significantly attenuated the expression of these inflammatory mediators compared with the PBS-treated group (*P* < 0.05, low-dose group versus PBS-treated group; *P* < 0.01, high-dose group versus PBS-treated group; *n* = 8). In addition, the levels of TNF-*α*, iNOS, MCP-1, RANTES, fractalkine, and ICAM-1 were more significantly reduced in the high-dose COS group than in the low-dose COS group (*P* < 0.05 in all paired comparisons; *n* = 8) (Figure 3).

### 3.4. IHC for the Effects of COS on the Expression of TNF-*α*, iNOS, MCP-1, RANTES, Fractalkine, and ICAM-1 in the ICB

**Figure 4:**
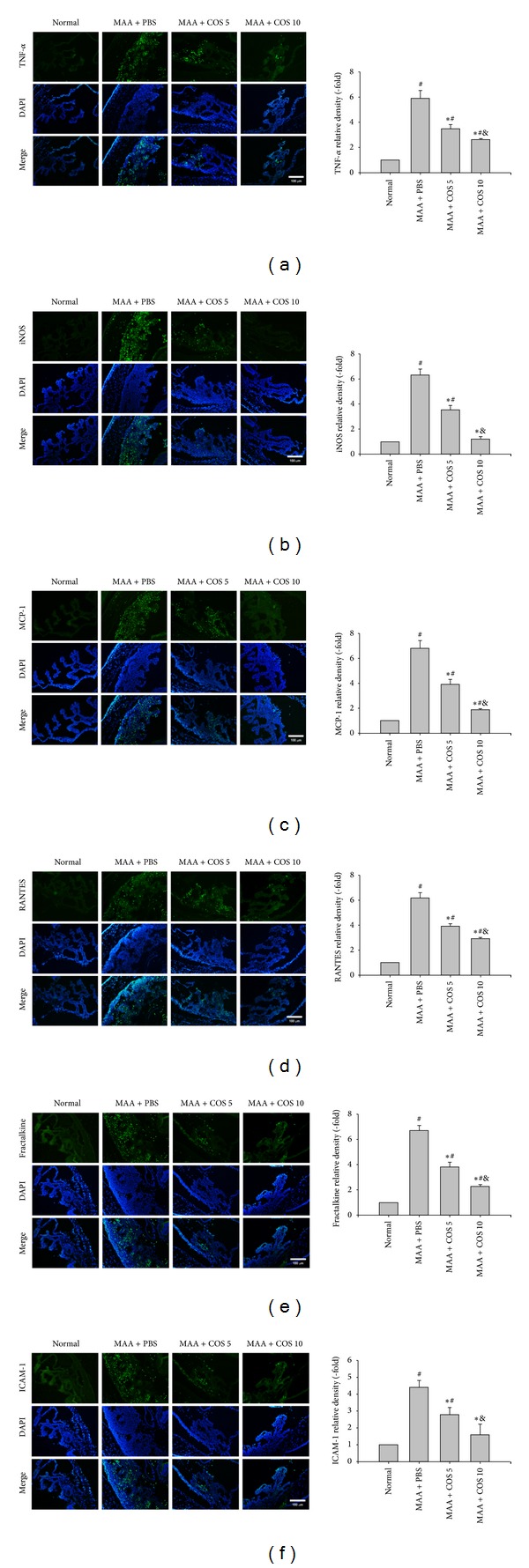
The evaluation of the expression levels of (a) TNF-*α*, (b) iNOS, (c) MCP-1, (d) RANTES, (e) fractalkine, and (f) ICAM-1 in the ICB at day 14 using IHC. For quantitation of immunostaining, we first determined the immunostaining index, which could be measured and calculated from the following formula: *∑*[(immunostaining density − threshold) × area  (pixels)]/total cell number. The relative density of immunostaining was defined as immunostaining index of PBS-treated, low-dose, or high-dose COS groups divided by immunostaining index of normal group. Treatment with low-dose or high-dose COS significantly decreased relative density of TNF-*α*, iNOS, MCP-1, RANTES, fractalkine, and ICAM-1 in the ICB, when compared with the PBS-treated group. The effects of decreased inflammatory mediators in the ICB were more noticeable in the high-dose COS group than in the low-dose group. There was little variation between the eyes in the same group. Original magnification 100x (^∗^ *P* < 0.05 compared with the PBS-treated group; ^#^ *P* < 0.05 compared with the normal group; ^&^ *P* < 0.05 compared with the low-dose group by Kruskal-Wallis *H* test with post hoc Dunn test; *n* = 3 for each group).

The immunohistochemical studies showed increased expression of TNF-*α*, iNOS, MCP-1, RANTES, fractalkine, and ICAM-1 in the ICB of the PBS-treated group at day 14. The low-dose and high-dose COS groups showed significantly decreased relative density of TNF-*α*, iNOS, MCP-1, RANTES, fractalkine, and ICAM-1 in the ICB, when compared with the PBS-treated group (*P* < 0.05 low-dose group versus PBS-treated group; *P* < 0.01, high-dose group versus PBS-treated group, *n* = 3). The relative density of TNF-*α*, iNOS, MCP-1, RANTES, fractalkine, and ICAM-1 was more reduced in the high-dose COS group than in the low-dose COS group (*P* < 0.05 high-dose group versus low-dose group, *n* = 3) (Figure 4).

### 3.5. ELISA for the Effects of COS on the Levels of TNF-*α*, NO, MCP-1, RANTES, and Fractalkine in the AqH

**Figure 5:**
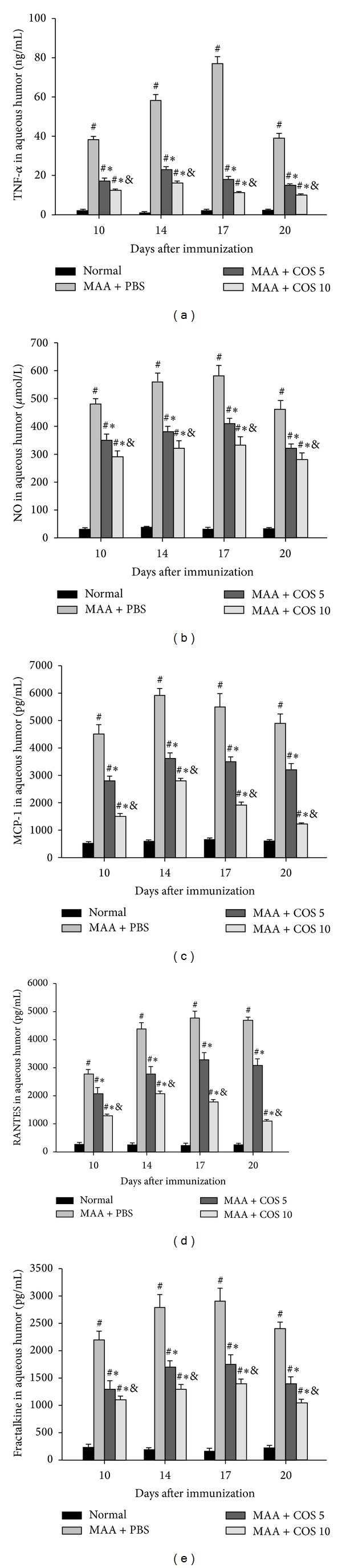
Quantification of the levels of (a) TNF-*α*, (b) iNOS, (c) MCP-1, (d) RANTES, and (e) fractalkine at days 10, 14, 17, and 20 in the AqH. Decreased expression levels of TNF-*α*, iNOS, MCP-1, RANTES, and fractalkine were observed in the low-dose or high-dose COS groups compared with the levels in the PBS-treated group. The AqH was pooled from one eye of five rats in each group. The data are expressed as the mean ± SD (^∗^ *P* < 0.05 compared with the PBS-treated group; ^#^ *P* < 0.05 compared with the normal group; ^&^ *P* < 0.05 compared with the low-dose group by Kruskal-Wallis *H* test with post hoc Dunn test; *n* = 5 for each group in each time point).

In the PBS-treated group, the levels of TNF-*α*, NO, MCP-1, RANTES, and fractalkine in the AqH were upregulated at days 10, 14, 17, and 20. Treatment with low-dose or high-dose COS significantly reduced the levels of TNF-*α*, NO, MCP-1, RANTES, and fractalkine in the aqueous humor at days 10, 14, 17, and 20 compared with the PBS-treated group (*P* < 0.05 low-dose group versus PBS-treated group; *P* < 0.01, high-dose group versus PBS-treated group, *n* = 8). In addition, the levels of TNF-*α*, MCP-1, fractalkine, RANTES, and NO in the aqueous humor were more reduced in the high-dose COS group than in the low-dose COS group (*P* < 0.05 in all comparisons, *n* = 8) (Figure 5).

### 3.6. Influence of COS on the Activation of NF-*κ*B in the ICB

**Figure 6:**
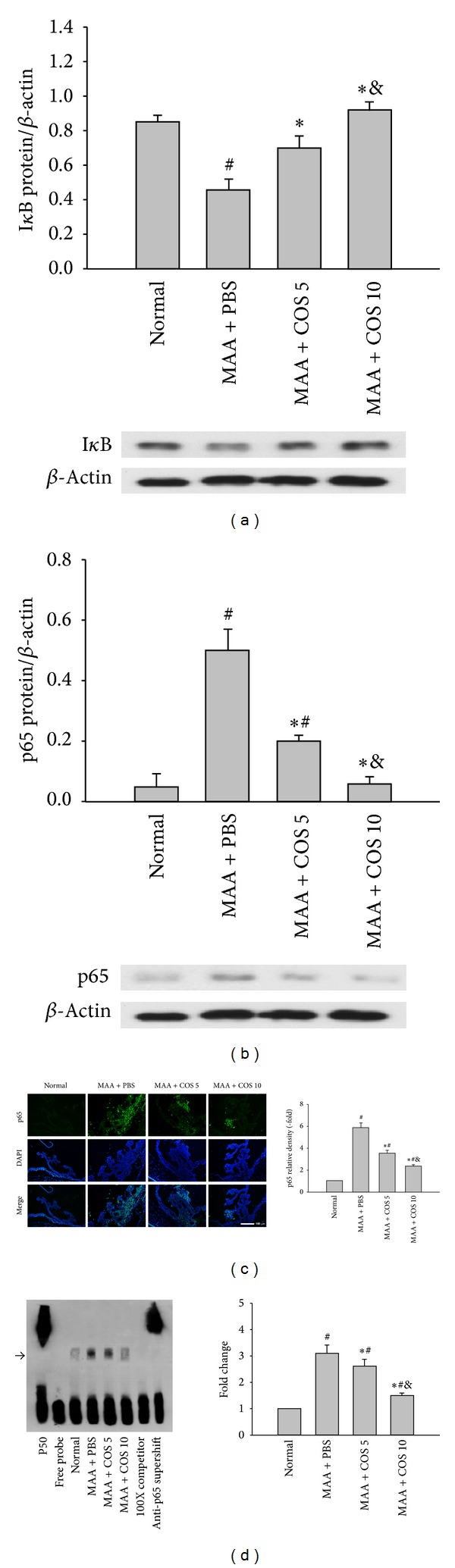
Effects of COS on the activation of NF-*κ*B in the ICB at day 14. Evaluation of (a) I*κ*B and (b) NF-*κ*B p65 in each group using western blot analysis. The Y scale represents the ratio of the I*κ*B or p65 blot density to the *β*-actin blot density (*n* = 8 for each group). (c) Immunohistochemical study of the expression of the NF-*κ*B p65 subunit in retinas. The images represent three rats in each group. (d) The NF-*κ*B/DNA binding activity in the ICB was measured using EMSA. “Fold change” was defined as optic density of the PBS-treated, low-dose, or high-dose COS group divided by optic density of the normal group (*n* = 5 for each group) (^∗^ *P* < 0.05 compared with the PBS-treated group; ^#^ *P* < 0.05 compared with the normal group; ^&^ *P* < 0.05 compared with the low-dose group by Kruskal-Wallis *H* test with post hoc Dunn test).

The levels of I*κ*B in the ICB were significantly reduced in the PBS-treated group at day 14. Treatment with COS significantly increased the expression of I*κ*B, especially in the high-dose COS group (*P* < 0.05 low-dose group versus PBS-treated group; *P* < 0.01, high-dose group versus PBS-treated group, *n* = 5) (Figure 6(a)). In contrast, the levels of p65 in the ICB were significantly increased in the PBS-treated group. Treatment with COS significantly decreased the expression of p65 in the ICB in a dose-dependent manner (*P* < 0.05 low-dose group versus PBS-treated group; *P* < 0.01, high-dose group versus PBS-treated group, *n* = 5) (Figure 6(b)).

Increased staining of the NF-*κ*B p65 subunit in the ICB was observed in the PBS-treated group at day 14. Treatment with low- and high-dose COS significantly reduced the relative density of p65 in the ICB (*P* < 0.05 low-dose group versus PBS-treated group; *P* < 0.01, high-dose group versus PBS-treated group, *n* = 3) (Figure 6(c)).

The PBS-treated group had increased activity of NF-*κ*B/DNA binding in the ICB at day 14. COS treatment significantly decreased the NF-*κ*B/DNA binding activity, and this inhibitory effect was especially prominent in the high-dose COS group (*P* < 0.05 low-dose group versus PBS-treated group; *P* < 0.01, high-dose group versus PBS-treated group, *n* = 5). Adding a 100-fold molar excess of the unlabeled NF-*κ*B probe completely inhibited the binding of the labeled probe to the NF-*κ*B/DNA complex (Figure 6(d)).

### 3.7. Effects of COS on Spleen-Derived Lymphocytes Chemotaxis toward AqH

**Figure 7:**
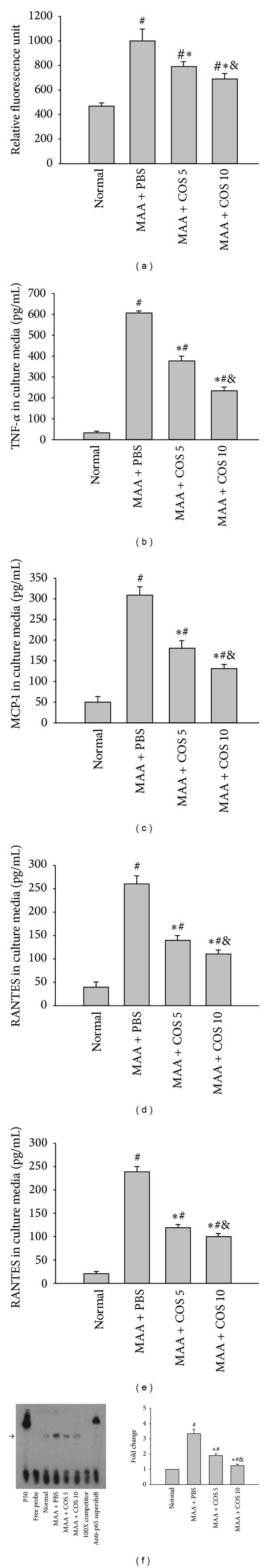
Effects of COS on spleen-derived lymphocyte chemotaxis, the secretion of inflammatory mediators, and the activation of NF-*κ*B. (a) Spleen-derived lymphocytes of each group were harvested and exposed to 10 *μ*g/mL MAA for 3 days. These cells were placed in the upper chamber of a QCM apparatus, and the aqueous humor of the same group was placed in the lower chamber of the QCM apparatus. After 24 hrs of incubation, the migrated cells were stained with a green fluorescent dye and subjected to ELISA and the relative fluorescence unit was calculated. The expression of (b) TNF-*α*, (c) MCP-1, (d) RANTES, and (e) fractalkine in the culture media of spleen-derived lymphocytes from each group was measured using ELISA. (f) The NF-*κ*B/DNA binding activity of spleen-derived lymphocytes from each group was measured using EMSA. “Fold change” was defined as optic density of the PBS-treated, low-dose, or high-dose COS group divided by optic density of the normal group (^∗^ *P* < 0.05 compared with the PBS-treated group; ^#^ *P* < 0.05 compared with the normal group; ^&^ *P* < 0.05 compared with the low-dose group by Kruskal-Wallis *H* test with post hoc Dunn test; *n* = 5 for each group).

The lymphocytes from the PBS-treated group exhibited markedly increased chemotaxis toward the aqueous humor obtained from the same group, more than that observed for the normal group. The chemotaxis was significantly decreased in the lymphocytes of the COS group, especially the high-dose COS group, compared with the PBS-treated group (*P* < 0.05 low-dose group versus PBS-treated group; *P* < 0.01, high-dose group versus PBS-treated group, *n* = 5) (Figure 7(a)).

### 3.8. Inflammatory Cytokine and Chemokine Secretion in the Culture Media by Spleen-Derived Lymphocytes after Stimulation with MAA In Vitro

When stimulated with MAA, the culture media of the lymphocytes from the PBS-treated group showed significantly increased expression of TNF-*α*, MCP-1, RANTES, and fractalkine compared with the culture media of the lymphocytes from the normal rats (*P* < 0.05 in all paired comparisons, *n* = 5). The levels of these inflammatory mediators were significantly reduced in the culture media of the lymphocytes from the COS group, especially in the high-dose COS group (*P* < 0.05 low-dose group versus PBS-treated group; *P* < 0.01, high-dose group versus PBS-treated group, *n* = 5) (Figures 7(b)–7(e)).

### 3.9. Influence of COS on the Activation of NF-*κ*B in Spleen-Derived Lymphocytes

The spleen-derived lymphocytes of the PBS-treated group had increased activity of NF-*κ*B/DNA binding. The lymphocytes of the COS-treated group showed markedly decreased NF-*κ*B/DNA binding activity, and this inhibitory effect was especially prominent in the high-dose COS group (Figure 7(f)).

## 4. Discussion

In this study, we demonstrated for the first time that COS effectively attenuated the clinical severity, diminished inflammation in the ICB, and reduced the expression and production of inflammatory cytokines and chemokines in rats with EAAU. We found that COS suppressed NF-*κ*B activation by inhibiting I*κ*B degradation and p65 translocation, which might contribute to the decreased proinflammatory cytokine and chemokine production observed in EAAU. In an in vitro study, we verified the results obtained in vivo by showing that sensitized spleen-derived lymphocytes of COS-treated group showed less chemotaxis toward the aqueous humor obtained from the same group. Moreover, the lymphocytes of the COS-treated group showed decreased NF-*κ*B activation and reduced secretion of the above proinflammatory mediators in the culture media. Our results suggested that COS most likely exerted anti-inflammatory effects in EAAU by the inhibition of NF-*κ*B activation and caused a reduction of the expression of inflammatory mediators, which in turn decreased trafficking and the recruitment of inflammatory cells to the inflammatory sites, leading to protection of the ICB from damage. Our results demonstrated the therapeutic potentials of COS in the treatment of acute anterior uveitis.

Significant leukocyte infiltration in the iris/ciliary body, especially by T lymphocytes, is the pathological hallmark of EAAU [16]. To selectively recruit leukocytes to the inflammatory sites, chemokines and cytokines are released to attract certain cell types; this is the crucial step in the pathogenesis of uveitis [17]. It is widely accepted that multiple cytokines, chemokines, and adhesion molecules, such as TNF-*α*, MCP-1, RANTES, fractalkine, iNOS, and ICAM-1, are implicated in the pathogenesis of uveitis [18–20]. TNF-*α* is an early proinflammatory cytokine that might activate macrophages and stimulate the synthesis of other cytokines, NO, and adhesion molecules, particularly ICAM-1, in acute uveitis [21]. MCP-1 RANTES and fractalkine are potent chemoattractants for T lymphocytes, monocytes, and NK cells, all of which are the infiltrating cells observed in the ICB in rats with EAAU [22–24]. In this study, we showed that COS could decrease the production of TNF-*α*, MCP-1, RANTES, fractalkine, and NO in the ICB in a dose-dependent manner, which might counteract the inflammatory response by inhibiting leukocyte recruitment into the eye. Several studies have demonstrated that limited numbers of sensitized and in vitro stimulated CD4 T-lymphocytes from spleen and lymph nodes could adoptively transfer EAAU to naive rats, suggesting a good accessibility of the anterior uveal target antigen to these cells [5, 25]. In in vitro study, we try to simulate the recruitment of sensitized lymphocytes to AqH and ICB in vivo by showing that the decreased expression of inflammatory mediators in AqH may result in less chemotaxis of sensitized T lymphocytes. In addition, we found that the secretion of TNF-*α*, MCP-1, RANTES, fractalkine, and NO was significantly reduced in the sensitized lymphocytes of the COS-treated rats. Taken together, our results demonstrated that COS reduced the expression of inflammatory mediators and decreased chemoattraction of T lymphocytes to the ICB. Moreover, COS could also abate the ability of the recruited lymphocytes to secrete chemokines and cytokines, further blocking the attraction of leukocytes to the inflammatory sites.

NF-*κ*B is a well-known transcription factor that could regulate gene expression involved in cellular proliferation, inflammation, and cell adhesion [26, 27]. Several studies have indicated that the expression of cytokines, chemokines, and adhesion molecules, such as TNF-*α*, MCP-1, RANTES, fractalkine, iNOS, and ICAM-1, is governed by NF-*κ*B [28–31]. We previously demonstrated a significant activation of NF-*κ*B in the ICB during EAAU, and the NF-*κ*B inhibitor pyrrolidine dithiocarbamate effectively reduced ocular inflammation in EAAU [23]. In this study, we demonstrated that COS could inhibit NF-*κ*B activation and decrease the expression and production of TNF-*α*, MCP-1, RANTES, fractalkine, iNOS, and ICAM-1, resulting in attenuated ocular inflammation. Previous studies have reported that COS exhibits anti-inflammatory activities by inhibiting NF-*κ*B activation in vitro and in vivo [32–35]. Yousef et al. showed that oral administration of 20 mg/kg/day of COS inhibited NF-*κ*B activation and the production of TNF-*α* and IL-6 in mouse models of experimental colitis and in human colonic epithelial cells (T84 cells) [36]. Wei et al. reported that COS at concentrations of 50 to 200 *μ*g/mL suppressed the production of NO in LPS-induced N9 murine microglial cells mediated by inhibiting the activation of NF-*κ*B and activator protein-1 [37]. In this study, we broadened the scope and demonstrated that COS treatment effectively inhibited NF-*κ*B activation and the production of multiple chemokines and cytokines in rat models of EAAU and in sensitized lymphocytes.

Movement of leucocytes through the vascular endothelium into inflammatory sites occurs in a series of stages, including rolling, arrest, firm adhesion, and transmigration [38]. ICAM-1 is a key molecule involved in leukocyte adhesion and transmigration. Several studies indicated that reduced expression of ICAM-1 results in attenuation of leukocyte adhesion and/or transmigration [39–41]. Previous studies have shown that increased expression of ICAM-1 is another important factor involved in the pathogenesis of EAAU [42]. In this study, we showed that the protein level of ICAM-1 in the ICB was upregulated and that COS significantly decreased the ICAM-1 expression in a dose-dependent manner. Our findings are consistent with a previous report by Li et al., who demonstrated that COS downregulated the expression of ICAM-1 by inhibiting the activation of NF-*κ*B in LPS-treated porcine iliac artery endothelial cells [43].

## 5. Conclusions

Our studies suggest that COS dampened the inflammatory damage by affecting diverse components of the inflammatory response, including chemokine and cytokine production and adhesion molecule expression in EAAU rats. The COS anti-inflammatory effects are most likely associated with the inhibition of NF-*κ*B activation. COS could be a promising agent for treating EAAU.

## Figures and Tables

**Table 1:** Summary of total number of animals at each time point in each group per experiment and days to perform the experiments.

Experiments	Number of animals at each time point in each group	Days after treatment
	(*n*)	
Clinical scores	10	Days 3, 7, 10, 14, 17, 20, 25, and 30
Histology	3	Day 14
Leukocyte counts	3	Days 10, 14, 17, and 20
Western blot analysis		
TNF-*α*, iNOS, MCP-1, RANTES, fractalkine, ICAM, p65, and i*κ*B	8	Day 14
Immunofluorescence		
TNF-*α*, iNOS, MCP-1, RANTES, fractalkine, and p65	3	Day 14
ELISA		
TNF-*α*, iNOS, MCP-1, RANTES, and fractalkine	5	Days 10, 14, 17, and 20
EMSA	5	Day 14
